# Integrative clinical and molecular analysis of advanced biliary tract cancers on immune checkpoint blockade reveals potential markers of response

**DOI:** 10.1002/ctm2.118

**Published:** 2020-08-12

**Authors:** Jingjing Li, Qing Wei, Xiaoying Wu, Jun Sima, Qi Xu, Mengmeng Wu, Fufeng Wang, Haibo Mou, Hanguang Hu, Jianguo Zhao, Da Li, Jinlin Hu, Lingnan Zhang, Xiu Zhu, Lei Chen, Cong Luo, Junrong Yan, Jiachen He, Yutong Ma, Yang Shao, Wei Wu, Jieer Ying

**Affiliations:** ^1^ Department of Abdominal Medical Oncology Cancer Hospital of the University of Chinese Academy of Sciences (Zhejiang Cancer Hospital) Institute of Cancer and Basic Medicine (IBMC) Chinese Academy of Sciences Hangzhou Zhejiang China; ^2^ Nanjing Geneseeq Technology Inc. Nanjing China; ^3^ Department of General Surgery Hangzhou Redcross Hospital Hangzhou China; ^4^ Department of Medical Oncology Shulan (Hangzhou) Hospital Hangzhou China; ^5^ Department of Medical Oncology Second Affiliated Hospital, Zhejiang University College of Medicine Hangzhou China; ^6^ Department of Oncology Shaoxing People's Hospital, Shaoxing Hospital of Zhejiang University Shaoxing China; ^7^ Department of Medical Oncology Sir Run Shaw Hospital, Zhejiang University School of Medicine Hangzhou China; ^8^ Department of Pathology Cancer Hospital of the University of Chinese Academy of Sciences (Zhejiang Cancer Hospital) Institute of Cancer and Basic Medicine (IBMC) Chinese Academy of Sciences Hangzhou Zhejiang China; ^9^ Radiology Department Cancer Hospital of the University of Chinese Academy of Sciences (Zhejiang Cancer Hospital) Institute of Cancer and Basic Medicine (IBMC) Chinese Academy of Sciences Hangzhou Zhejiang China

**Keywords:** biliary tract cancer, immune checkpoint blockade, NGS, predictive biomarkers

## Abstract

**Background:**

While there have been encouraging preliminary clinical results for immune checkpoint inhibitors (ICIs) in BTCs, it remains a challenge to identify the subset of patients who may benefit. In this study, we evaluated the efficacy of ICI treatment in patients with advanced BTCs, and explored potential biomarkers that are predictive of response.

**Methods:**

The study enrolled 26 patients with advanced microsatellite stable BTCs (15 with gallbladder cancers [GCs] and 11 with intrahepatic cholangiocarcinoma [ICCs]) who received ICI treatment. Targeted next‐generation sequencing (NGS) was performed on tumor tissue samples collected from 17 patients. Clinical and genomic characteristics were assessed for the correlation with clinical outcome.

**Results:**

Analysis of the baseline clinical characteristics showed that performance score (PS) of 0 was associated with a better prognosis than PS of 1 (HR = 1.08 × 10^9^; 95% CI, 0∼Inf; *P* = .002). No significant correlations were found between clinical outcome and inflammation‐related indicators. NGS profiling of the available tumor tissues, revealed largely non‐overlapping somatic alterations between GCs and ICCs. Mutations in *LRP1B* (HR = 0.26; 95% CI, 0.06‐1.21; *P* = .067), *ERBB2* (HR = 0.15; 95% CI, 0.02‐1.19; *P* = .04), or *PKHD1* (HR < 0.01; 95% CI, 0‐Inf; *P* = .04) showed strong association with increased progression‐free survival (PFS) benefit. Subsequent analysis showed that alterations in the RTK‐RAS pathway were associated with improved outcomes (HR = 0.12; 95% CI, 0.02‐0.63; *P* = .003). Tumor mutation burden (TMB) was higher in patients with GC than those with ICC, and was associated with *LRP1B* mutations (*P* = .032). We found that patients with 19q amplification (19q Amp) and 9p deletion (9p Del) had poor PFS outcome (19q Amp, HR = 15.4; 95% CI, 2.7‐88.5; *P* < .001; 9p Del; HR = 4.88 × 10^9^; 95% CI, 0‐Inf; *P* < .001), while those with chromosomal instability derived PFS benefit (HR = 0.24; 95% CI, 0.05‐1.17; *P* = .057).

**Conclusion:**

Our study identified several potential clinical and genomic features that may serve as biomarkers of clinical response to ICIs in advanced BTCs patients. A larger sample size is required for further verification.

## Graphical abstract

Mutational profiles of BTCs showed distinct pattern with GCs and ICCs. Immunotherapy response was associated with several individual somatic gene mutations and RTK‐RAS pathway alterations. Patients with high chromosome instability tended to have improved PFS outcome from ICI treatment. Chromosome arm‐level SCNAs, 19q amplification, and 9p deletion showed associations with poor prognosis.

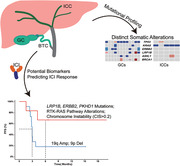

## 1 INTRODUCTION

Biliary tract cancers (BTCs), including cholangiocarcinoma and gallbladder cancers (GCs), are uncommon malignancies characterized by a high mortality rate.^1^, ^2^ The majority of patients present with advanced disease at initial diagnosis. Systemic therapy or chemoradiation remains the standard first‐line strategy for patients with unresectable or metastatic BTCs.^3^, ^4^ However, given their poor prognosis and almost inevitable resistance to chemotherapy, alternative therapeutic options are urgently needed.

Immune checkpoint blockade has demonstrated substantial benefits in multiple disease settings.^5^, ^6^, ^7^, ^8^ However, the role of immunotherapy in advanced BTCs patients remains to be explored. While cancers with deficient mismatch repair (dMMR) or microsatellite instability‐high (MSI‐H) are likely to benefit from anti‐PD‐1 inhibitor (eg, pembrolizumab [Keytruda]), BTCs are infrequently MSI‐H or dMMR.^9^ In the KEYNOTE‐028 phase Ib basket trials across 20 advanced solid tumor types that include advanced BTCs, patients with high tumor mutation burden (TMB) and/or PD‐L1 expression demonstrated higher response rates and better progression‐free survival (PFS).^10^ Follow‐up studies on patients with advanced BTCs treated with immunotherapy in the KEYNOTE‐158 (phase II) and KEYNOTE‐028 (phase Ib) trials present preliminary evidence showing durable clinical outcome regardless of PD‐L1 expression.^11^ At present, multiple early‐phase clinical trials are ongoing to evaluate the efficacy of immunotherapy alone or in combination with other therapies in advanced BTCs.^12^

Selecting the dominant population is the key problem of immunotherapy for BTCs. NGS currently considered as a relatively advantageous technology to find the dominant population for immunotherapy. Some studies suggested that high TMB (TMB‐H) and MSI‐H showed relationship with higher responding rates and durability to immune checkpoint inhibitors (ICIs) for BTCs.^13^, ^14^ One case report showed that insertions and deletions (indels) might be a new predictive biomarker of response to ICIs in patients suffering from intrahepatic cholangiocarcinomas (ICCs) beside the status of PD‐L1, TMB, and MSI.^15^ Recently, the whole‐exome sequencing in one phase II study shown that TMB, tumor neoantigen burden (TNB), and the mutation of *RYR2*, *MUC4*, and *APOB* could predict the efficacy of nivolumab plus gemcitabine and cisplatin for BTCs.^16^ However, no other extensive analyses of genomic correlates of immunotherapy response have been carried out in BTCs.

In this study, by evaluating the clinical and molecular characteristics of 26 advanced BTCs patients, we aimed to evaluate the therapeutic effect of anti‐PD‐1 inhibitors, as monotherapy or in combination with other treatment regimens, and to explore potential biomarkers that may be predictive of immunotherapy outcome.

## 2 MATERIALS AND METHODS

### 2.1 Study design and participants

This single‐center study was performed at the Cancer Hospital of the University of Chinese Academy of Sciences (Zhejiang Cancer Hospital) with approval of local human research ethics committee. The study included 26 unresectable or metastatic BTCs patients who received immune checkpoint inhibitors (ICIs) therapy between December 2018 and September 2019. All patients were asked to provide informed consents in accordance with institutional regulations. Clinicopathological information, including sex, age, tumor location, Eastern Cooperative Oncology Group (ECOG) performance status (PS), therapeutic approach, lines of treatment, metastasis, serum inflammatory factors (eg, C‐reactive protein [CRP], neutrophil‐to‐lymphocyte ratio [NLR], lactate dehydrogen [LDH], systemic immune‐inflammation index [SII], prognostic index [PI], Glasgow Prognostic Score [GPS], prognostic nutritional index [PNI]), and serum tumor markers, such as carbohydrate antigen 125 (CA125; cutoff value: 5 ng/mL), carcinoembryonic antigen (CEA; cutoff value: 5 ng/mL), and carbohydrate antigen 19‐9 (CA19‐9; cutoff value: 30 U/mL) were reviewed, retrospectively.

### 2.2 Library construction and targeted enrichment

NGS was conducted in a centralized testing center (Nanjing Geneseeq Technology Inc.). Sample processing, library construction, and targeted‐enrichment were performed according to the methods as previously described.^17^ Briefly, DNA from the white blood cells (WBCs) or formalin‐fixed and paraffin‐embedded (FFPE) was extracted, quantified, and quality‐evaluated, respectively. Genomic DNA from WBCs was used as the normal control.

Libraries were constructed as previously described.^18^ Briefly, the extracted DNA was sheared into fragments, and end repair, A‐tailing, and adaptor ligation were further performed. DNA Libraries were then amplified and purified. Next, customized xGen lockdown probes panel (containing 425 predefined cancer‐related genes) were used for selective enrichment. Then, the captured libraries were amplified, purified, and quantified.

### 2.3 Library sequencing and bioinformatics analysis

Enriched libraries were sequenced on the HiSeq4000 platform (Illumina). Data were sequentially analyzed by validated bioinformatics process.^19^ Further, SNPs and indels were called with a variant allele frequency (VAF) cutoff as 0.5% by VarScan2.^20^ dbSNP and the 1000 Genome project databases were used to remove the common variants. Gene fusions and copy number variations (CNVs) were identified by FACTERA^21^ and ADTEx,^22^ respectively. The log2 ratio cutoff for copy number gain and loss was defined as 2.0 and 0.6, respectively. Arm‐level somatic copy number alterations (SCNAs) were analyzed by FACETS^23^ with a 0.2 drift cutoff for unstable joint segments. Chromosome instability score (CIS) was defined as previously described.^24^ TMB was calculated according to Fang's study.^5^ TMB‐H was defined as TMB above the top 33% of the cohort, and the rest lower than top 33% was defined as low TMB (TMB‐L).

### 2.4 Statistical analysis

Quantitative data are displayed as median (range) or number of patients (percentage). Proportion comparisons between groups were carried out with the Fisher's exact test. Survival analysis was performed using Kaplan‐Meier curves, and the *P*‐value was determined with the log‐rank test, and hazard ratios (HRs) were calculated by Cox proportional hazards model. A two‐sided *P*‐value of <.05 was considered as a significant value for all tests unless indicated otherwise. Univariate analysis was performed to study the associations between different variables and PFS, and the results were presented as HRs and the 95% confidence intervals (CIs). All analyses were performed with R 3.4.0.

## 3 RESULTS

### 3.1 Patient characteristics

Baseline clinicopathological features of the study cohort with advanced unresectable BTCs are summarized in Table 1 (median age 62.5 years, range 46‐73; 34.6% of patients were females). All patients showed an ECOG PS of 0‐1. Of all the patients, 15 had gallbladder carcinomas (GCs), 11 had intrahepatic cholangiocarcinomas (ICCs), and none had extrahepatic cholangiocarcinoma. Lymph nodes and liver metastases were common, with incidence rates of 76.9% and 61.5%, respectively. Fifteen patients had no prior systemic therapies, and the other 11 patients had received at least one systemic therapy and the majority (25/26, 96.2%) of them received combination therapy that involved either chemotherapy (84.6%) or anti‐angiogenic agents (11.5%). Only one patient received ICI monotherapy.

**TABLE 1:** Clinical characteristics of the study population (n = 26)

Characteristics	Number (%)
Age, years (median)	62.5 (46‐73)
≤65	16 (61.5%)
>65	10 (38.5%)
Sex	
Female	9 (34.6%)
Male	17 (65.4%)
Location	
Gallbladder	15 (57.7%)
Intrahepatic	11 (42.3%)
ECOG PS	
0–1	26 (100%)
2–3	0 (0%)
Stage	
I‐III	0 (0%)
IV	26 (100%)
Combined therapy	
Mono	1 (3.9%)
Combined with Chemo	22 (84.6%)
Combined with Antiangiogenic drugs	3 (11.5%)
Cycle of treatment (range)	4 (1‐24)
≥4	16 (61.5%)
<4	10 (38.5%)
Line of treatment	
1	15 (57.7%)
≥2	11 (42.3%)
Number of metastasis organs	
1	12 (46.2%)
≥2	14 (53.8%)
Organ of metastasis	
Lymph node	20 (76.9%)
Peritoneal metastasis	6 (23.1%)
Liver	16 (61.5%)
Lung	2 (7.7%)
Response	
Confirmed Objective response	7 (26.9%)
Confirmed Disease control rate	19 (73.1%)
Best overall response	
Complete response	1 (3.8%)
Partial response	6 (23.1%)
Stable disease	12 (46.2%)
Progressive disease	6 (23.1%)
NE (Not Evaluable)	1 (3.8%)

### 3.2 Efficacy and safety

**FIGURE 1:**
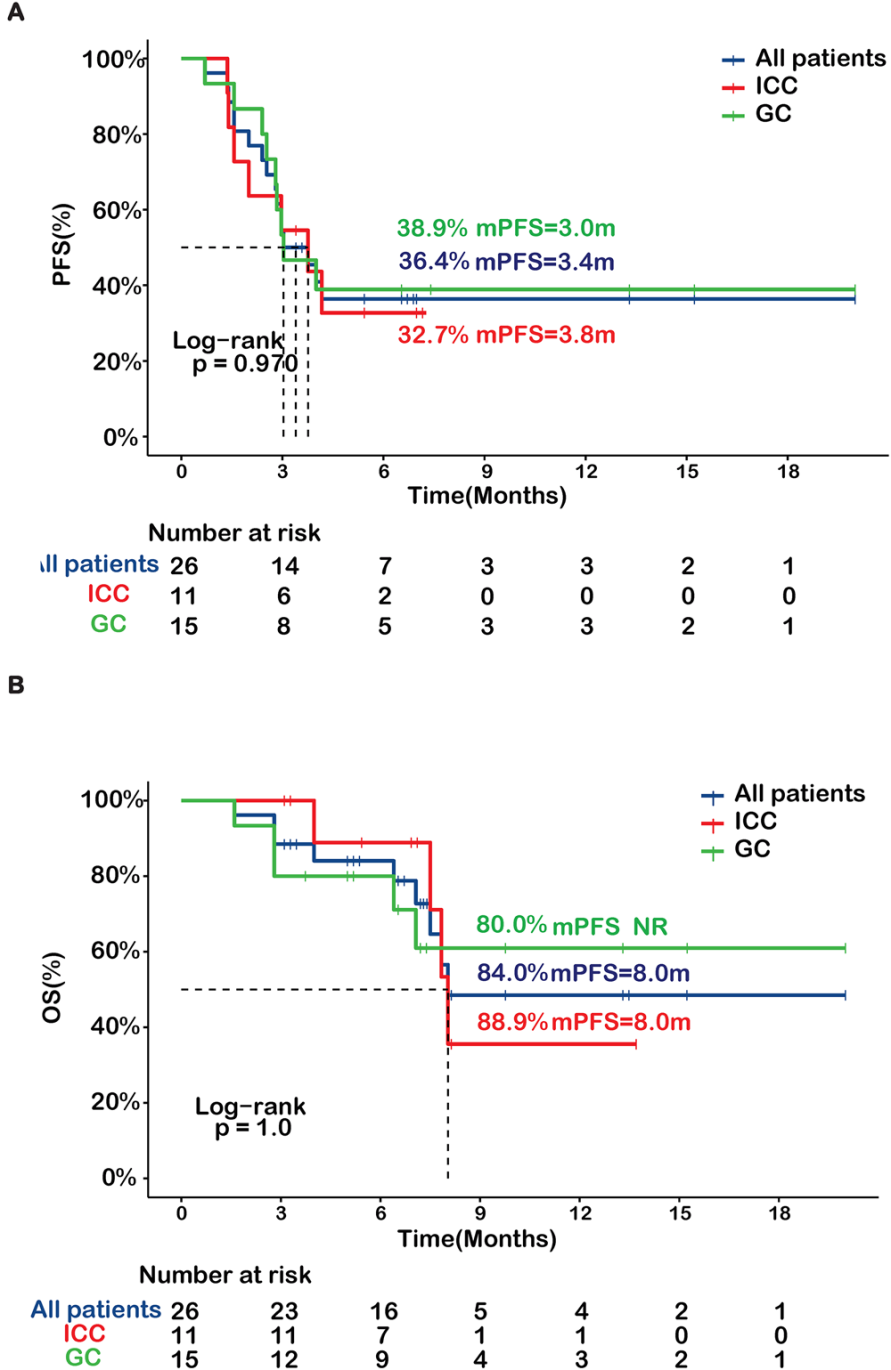
PFS (**A**) and OS (**B**) of the GC, ICC, and all BTCs in this study

As of December 21, 2019, 16 (61.5%) events of disease progression and nine (34.6%) deaths had occurred. The median PFS (mPFS) was 3.4 months, with a 6‐month PFS rate of 36.4%. No difference in PFS was found between GCs and ICCs (Figure 1A). The median overall survival (OS) was 8.0 months with a 6‐month OS rate of 84.0%. Similarly, OS showed no difference between GCs and ICCs (Figure 1B). Among all 26 patients, one (3.8%) patient achieved complete response (CR), and six (23.1%) patients achieved partial response (PR), which corresponded to an objective response rate (ORR) of 26.9% (Table 1). Most patients (96.1%) developed treatment‐related adverse events (AEs), with grade I/II and III/IV AEs occurring in 80.8% and 30.8% of the patients, respectively. Most common grade III/IV AEs included neutropenia (19.2%), thrombocytopenia (11.5%), anemia (7.7%), anorexia (7.7%), increased aspartate aminotransferase (AST; 7.7%), and alanine aminotransferase (ALT; 7.7%; Table S1)

### 3.3 Association between clinical features and response to immunotherapy

Associations analysis between clinicopathological characteristics and clinical outcomes revealed a PFS improvement in male patients compared with female patients (mPFS, 2.5 months vs not reached [NR]; HR = 0.32; 95% CI, 0.10∼1.00; *P* = .016). In addition, patients with performance score (PS) of 0 demonstrated improved PFS comparing with those with PS of 1 (mPFS, NR vs 2.9 months; HR = 1.08 × 10^9^; 95% CI, 0∼Inf; *P* = .002). Further correlation analysis demonstrated a moderate correlation between gender and PS scores (Spearman coefficient, −0.453; two‐sided *P* = .02) with majority of male patients presenting relative better performance, potentially resulting in this gender‐biased efficacy difference. Other clinical features, such as age, BTC subtypes, treatment lines, number of metastases, and site of metastasis were not significantly associated with the efficacy of ICI (Table 2). Furthermore, as previous studies suggested, chronic inflammation may play an important role in the carcinogenesis of BTC,^25^ we evaluated the relationships between the systemic inflammation status and immunotherapy outcome, by profiling the set of systemic inflammation markers, including LDH, CRP, NLR, SII, GPS, PI, and PNI. However, none of these markers showed significant correlations with PFS outcome (Table 2). In addition, assessment of tumor markers, including CEA, CA125, and CA199, also revealed no association with immunotherapy outcome (Table 2).

**TABLE 2:** Univariate Cox regression analysis of PFS with clinical characteristics, signal pathways and chromosome arm‐level SCNAs in all patients (n = 26), BTCs (n = 17), or GCs (n = 12) underwent sequencing

Characteristics	mPFS	HR (95%CI)	*P*‐value
**Clinical information (All patients, n = 26)**			
Age, years (median) (≤ 65 vs > 65)	3.8 versus 2.7	1.28 (0.46∼3.54)	0.638
Sex (Female vs Male)	2.5 versus NR	0.32 (0.10∼1.00)	0.016
Tumor site (Gallbladder vs Intrahepatic)	3.0 versus 3.8	1.12 (0.42∼3.02)	0.821
ECOG PS (0 vs 1)	NR versus 2.9	1.08 × 10^+09^ (0∼Inf)	0.002
Line of treatment (1 vs ≥ 2)	3.0 versus 4.0	0.74 (0.267∼2.03)	0.554
Number of metastasis organs (1 vs ≥ 2)	2.8 versus 4.2	0.42 (0.15∼1.16)	0.085
Site of metastasis (Lymph node metastasis only vs Visceral metastasis (With or without lymph node metastasis))	2.8 versus 3.4	1.40 (0.40∼4.92)	0.603
CEA (< 5ug/L vs ≥5ug/L)	4.2 versus 3.8	0.97 (0.34∼2.78)	0.948
CA125 (< 35 U/L vs ≥35 U/L)	NR versus 3.8	2.03 (0.63∼6.51)	0.224
CA199 (< 37 U/L vs ≥37 U/L)	3.0 versus 4.0	0.93 (0.31∼2.79)	0.899
LDH (< 245 U/L vs ≥245 U/L)	3.8 versus 4.2	1.13 (0.39∼3.26)	
CRP (< 10 mg/L vs ≥10 mg/L)	NR versus 3.0	1.73 (0.60∼5.00)	0.308
NLR (< 3 vs ≥3)	3.4 versus 4.2	0.70 (0.215∼2.24)	0.54
dNLR (< 2.5 vs ≥2.5)	3.8 versus 4.0	1.13 (0.394∼3.22)	0.824
PLR (< 150 vs ≥150)	3.8 versus 3.5	0.80 (0.28∼2.31)	0.679
SII (< 800 vs ≥800)	3.8 versus 4.0	0.91 (0.32∼2.61)	0.864
GPS (0 vs 1&2)	NR versus 3.0 versus 3.4	1.39 (0.72∼2.67)	0.326
PI (0 vs 1)	NR versus 3.9	1.73 (0.60∼5)	0.308
PNI (0 vs 1)	12.1 versus 3.8	1.58 (0.49∼5.09)	0.443
**Signal Pathway (BTCs, n = 17)**			
RTK‐RAS (Altered vs WT)	4.0 versus 2.4	0.12 (0.02∼0.63)	0.003
TP53 (Altered vs WT)	2.9 versus 4.2	1.66 (0.48∼5.69)	0.417
Cell Cycle (Altered vs WT)	3.0 versus 3.4	1.61 (0.47∼5.5)	0.446
PI3K (Altered vs WT)	NR versus 3.0	0.45 (0.10∼2.07)	0.289
WNT (Altered vs WT)	4.0 versus 3.0	0.74 (0.21∼2.54)	0.626
TGF‐Beta (Altered vs WT)	3.0 versus 3.4	1.49 (0.44∼5.11)	0.522
NOTCH (Altered vs WT)	3.8 versus 3.0	0.90 (0.19∼4.19)	0.896
**Signal Pathway (GCs, n = 12)**			
RTK‐RAS (Altered vs WT)	NR versus 2.4	0.11 (0.02∼0.69)	0.005
TP53 (Altered vs WT)	2.9 versus 3.0	1.45 (0.28∼7.50)	0.656
Cell Cycle (Altered vs WT)	2.5 versus 3.0	2.00 (0.38∼10.50)	0.403
PI3K (Altered vs WT)	NR versus 3.0	0.46 (0.09∼2.39)	0.345
WNT (Altered vs WT)	3.0 versus 2.9	0.62 (0.12∼3.23)	0.567
TGF‐Beta (Altered vs WT)	2.5 versus NR	3.81 (0.84∼17.30)	0.064
MYC (Altered vs WT)	NR versus 2.9	3.52 × 10^‐09^ (0∼Inf)	0.140
NOTCH (Altered vs WT)	2.8 versus 3.0	0.80 (0.10∼6.69)	0.837
HIPPO (Altered vs WT)	NR versus 2.9	3.52 × 10^‐09^ (0∼Inf)	0.14
**Chromosome arm‐level SCNA (BTCs, n = 17)**			
20q_Amp (Yes vs No)	3.6 versus 3.0	0.90 (0.27∼2.96)	0.856
5p_Amp (Yes vs No)	3.0 versus 3.4	0.91 (0.24∼3.43)	0.889
18p_Amp (Yes vs No)	2.0 versus 3.8	2.57 (0.67∼9.88)	0.155
19q_Amp (Yes vs No)	2.0 versus 4.2	15.40 (2.69∼88.50)	7.84E‐05
20p_Amp (Yes vs No)	2.9 versus 3.0	1.55 (0.405∼5.89)	0.521
7p_Amp (Yes vs No)	4.2 versus 3.0	0.50 (0.11∼2.31)	0.361
10q_Del (Yes vs No)	NR versus 3.0	0.37 (0.05∼2.93)	0.329
19p_Del (Yes vs No)	1.6 versus 3.4	1.88 (0.40∼8.83)	0.414
3p_Del (Yes vs No)	NR versus 3.0	0.43 (0.05∼3.37)	0.406
9p_Del (Yes vs No)	1.6 versus 4.0	4.88 × 10^09^ (0∼Inf)	2.94E‐06
**Chromosome arm‐level SCNA (GCs, n = 12)**			
20q_Amp (Yes vs No)	3.0 versus 3.0	1.07 (0.24∼4.82)	0.928
5p_Amp (Yes vs No)	3.0 versus 3.0	0.97 (0.22∼4.36)	0.967
18p_Amp (Yes vs No)	2.5 versus 3.0	2.00 (0.38∼10.50)	0.403
19q_Amp (Yes vs No)	2.4 versus NR	8.93 (1.44∼55.30)	0.005
20p_Amp (Yes vs No)	1.6 versus 3.0	1.13 (0.13∼9.43)	0.913
7p_Amp (Yes vs No)	2.8 versus 3.0	0.71 (0.08∼5.92)	0.749
10q_Del (Yes vs No)	NR versus 3.0	0.41 (0.05∼3.40)	0.392
19p_Del (Yes vs No)	1.6 versus 3.0	1.13 (0.13∼9.43)	0.913
9p_Del (Yes vs No)	2.0 versus 3.0	3.15 × 10^09^ (0∼Inf)	0.0002

Abbreviations: CEA, carcinoembryonic antigen; CA125, carbohydrate antigen 125; CA199, carbohydrate antigen 199; LDH, lactate dehydrogenase; CRP, C‐reactive protein; NLR, neutrophil lymphocyte ratio; dNLR, derived neutrophils/(leukocytesminus neutrophils) ratio; PLR, platelet to lymphocyte ratio; SII, systemic immune‐inflammation index; GPS, Glasgow prognostic score; PI, prognostic index; PNI, prognostic nutritional Index; CI, confidence interval; SCNA, somatic copy number alteration; WT, wild‐type.

### 3.4 Individual somatic gene alterations associated with immunotherapy response

**FIGURE 2:**
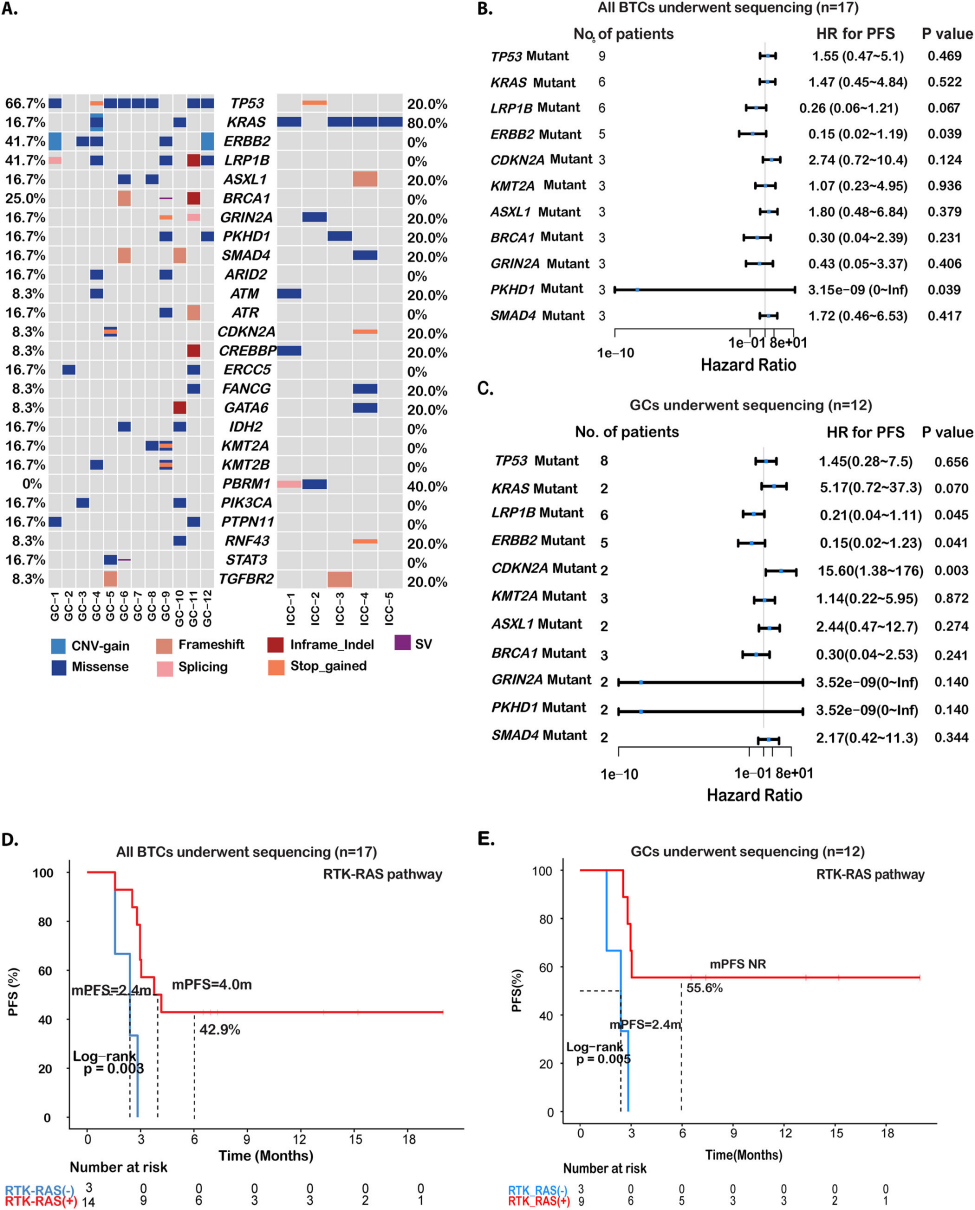
Mutational spectrum of advanced BTCs and genetic correlates of immunotherapy outcome. **A,** Comparison of mutation profiles between GCs and ICCs. The top 26 genes of the NGS cohort (n = 17) is shown. **B,C**, Forest plot presenting hazard ratios (HRs) of PFS comparing various subgroups with and without single‐gene variations in BTCs (n = 17) or GCs (n = 12), respectively. **D,E,** Kaplan‐Meier estimates of PFS comparing the subgroups with and without alterations in RAS‐RTK pathway of BTCs (n = 17) or GCs (n = 12), respectively

Next, we sought to explore the molecular features that correlate with immunotherapy response. Tumor tissue samples were available from 17 patients, including 12 patients with GCs and five with ICCs, which were subjected to targeted NGS using a 425‐cancer‐gene panel. Even with the modest sample size, the differences in mutational profiles of the GC and ICC samples were evident (Figure 2A). Specifically, *TP53* (66.7%), *ERBB2* (41.7%), *LRP1B* (41.7%), and *BRCA1* (25%) were among the top altered genes in GCs. All except one of the ICCs harbored *KRAS* mutations (80%), but none carried alterations in *ERBB2*, *LRP1B*, or *BRCA1*. Further, BTC patients with mutations in *LRP1B*, *ERBB2*, or *PKHD1* showed prolonged PFS than wild‐type patients (Figure 2B). Patients with *LRP1B* mutations had an mPFS that was not reached (NR) and a 6‐month PFS rate of 66.7%, compared with an mPFS of 3.0 months and a 6‐month PFS rate of 18.2% in the rest of the patients (HR = 0.26; 95% CI, 0.06‐1.21; *P* = .067; Figures 2B and 6A). Of the five *ERBB2* mutations, two were copy number gain and three were missense mutations of indeterminate effect. In contrast to the poor outcome in non‐small‐cell lung cancer (NSCLC) with *ERBB2* alterations,^26^ *ERBB2‐*mutated BTC patients showed improved PFS compared with those of wild‐type *ERBB2* (mPFS, NR vs 3.0 months, HR = 0.15; 95% CI, 0.02‐1.19; *P* = .039; 6‐month PFS rate = 80.0% vs 16.7%; Figures 2B and 6B). Two *ERBB2*‐positive patients (one with CNV gain and one with missense mutation) also carried mutations in *PKHD1*. At data cutoff, none of the patients with *PKHD1* mutations progressed, compared with a 6‐month PFS rate of 21.4% in *PKHD1* wild‐type patients (mPFS = NR vs 3.0 months, HR < 0.001; 95% CI, 0‐Inf; *P* = .039; Figures 2B and 6C). Consistent with their associations with longer PFS, patients with *LRP1B* (ORR = 50.0% vs 27.3%; Figure 6D), *ERBB2* (ORR = 40.0% vs 33.3%; Figure 6E), or *PKHD1* (66.7% vs 28.6%; Figure 6F) alterations were also more likely to experience objective response, although no statistical significance could be established given the limited sample size.

Sub‐analysis of the GC cohort revealed similar PFS benefit associated with *LRP1B* mutations (mPFS, NR vs 3.0 months; 6‐month PFS rate, 66.7% vs 16.7%; HR = 0.21; 95% CI, 0.04‐1.11; *P* = .045; Figures 2C and 6G) and *ERBB2* alterations (mPFS = NR vs 2.8 months, HR = 0.15; 95% CI, 0.02‐1.23; *P* = .041; 6‐month PFS rate, 80.0% vs 14.3%; Figures 2C and 6H). On the other hand, GC patients with *PKHD1* mutations did not show significant improvement of PFS over those without *PKHD1* mutations (*P* = .140). In addition, we found that GC patients with *CDKN2A* mutations showed worse mPFS than those with WT *CDKN2A* (mPFS = 3.0 vs 2.1 months, HR = 15.60; 95% CI, 1.38‐176; *P* = .003; 6‐month PFS rate 0% vs 50.0%; Figures 2C and 6I).

### 3.5 Signaling pathways associated with clinical outcomes to immunotherapy

Multiple oncogenic signaling pathways have demonstrated implications in the response or resistance to ICIs treatments.^27^ Thus, somatic mutations and copy number variations in genes in different signaling pathways were evaluated for their associations with patient outcomes. The common signaling pathways altered in the BTC cohort included the RTK‐RAS, TP53, cell cycle, PI3K, WNT, TGF‐β, and Notch pathways (Table 2). We found that patients with genetic alterations in the RTK‐RAS pathway derived greater PFS benefit from ICI treatment (mPFS = 4.0 vs 2.4 months; HR = 0.12; 95% CI, 0.02‐0.63; *P* = .003; Table 2 and Figure 2D). The ORR in patients with altered RAS‐RTK pathway was 42.8%, while none of the patients with unaffected RAS‐RTK pathway responded to ICI treatment (Figure S1A).

Among the commonly altered pathways in GCs, including the RTK‐RAS, TP53, cell cycle, PI3K, WNT, TGF‐β, MYC, Notch, and HIPPO pathways, we also found that patients with altered RTK‐RAS signaling had greater PFS benefit (mPFS, NR vs 2.4 months, HR = 0.11; 95% CI, 0.02‐0.69; *P* = .005; Figure 2E and Table 2), as well as a higher ORR (44.4% vs 0%) from ICI treatment (Figure S1D).

### 3.6 Chromosome arm‐level SCNAs correlated with immunotherapy benefit

**FIGURE 3:**
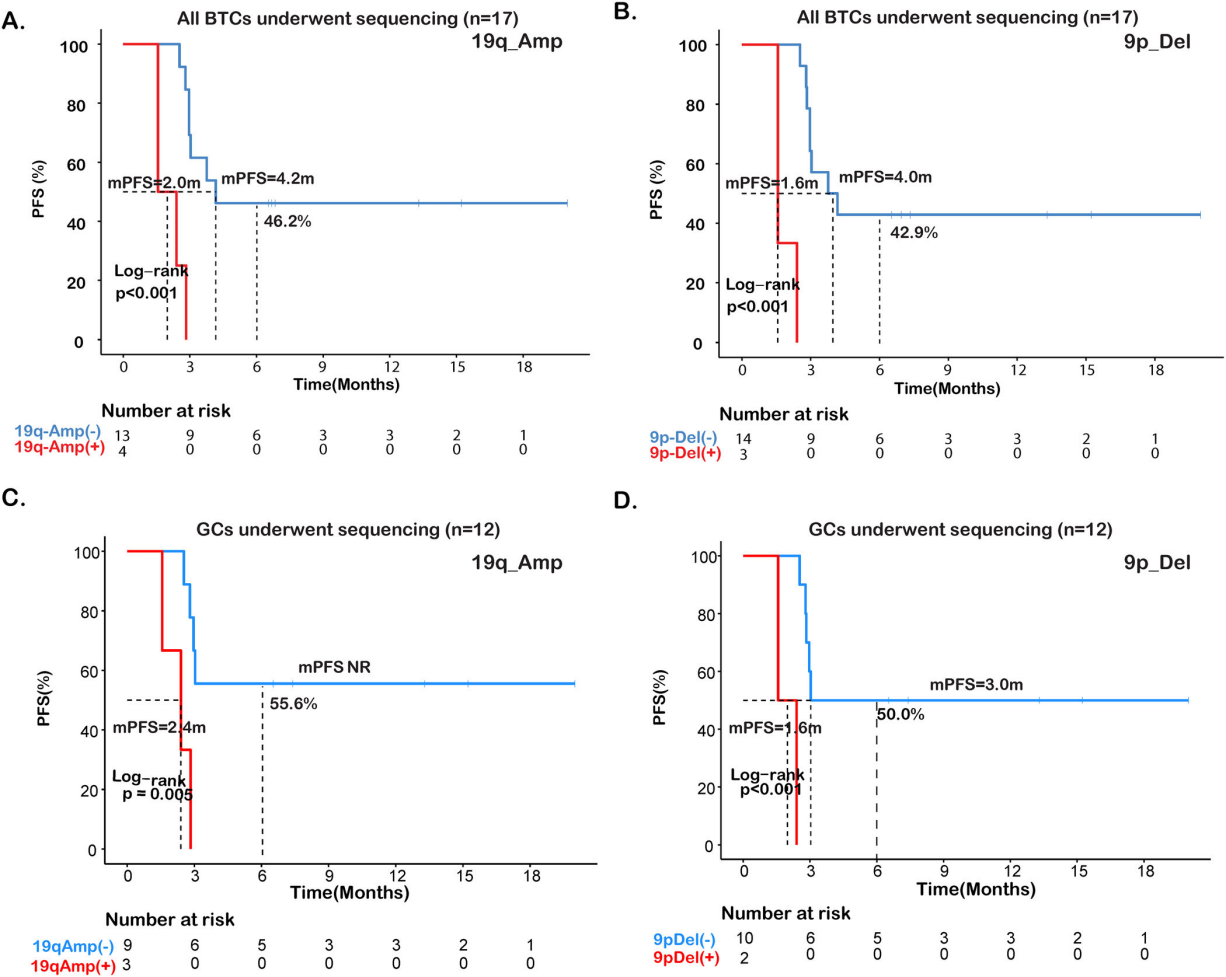
Associations of arm‐level SCNAs with immunotherapy outcome. Kaplan‐Meier estimates of PFS comparing the subgroups with and without alterations in (**A**) chromosome arm 19q amplification (19q_Amp) or (**B**) 9p deletion (9p_Del) of BTCs (n = 17), and in (**C**) chromosome arm 19q amplification (19q_Amp) or (**D**) 9p deletion (9p_Del) of GCs (n = 12)

Chromosome arm‐level SCNAs often involve dosage changes of specific gene sets that modulate tumor growth or immune response, which are selected for during tumor evolution.^28^ In all BTC patients, analysis of arm‐level SCNAs revealed 19q amplification (19q_Amp) (HR = 15.40, 95% CI, 2.69‐88.5; *P* < .001; Figure 3A and Table 2) and 9p deletion (9p_Del) (HR = 4.88e9; 95% CI, 0‐Inf; *P* < .001; Figure 3B and Table 2) that showed associations with poor prognosis. Similarly, a lower ORR was observed for patients with 19q_ Amp (0% vs 46.2%; Figure S1B) or those with 9p Del (0% vs 42.8%; Figure S1C).

Subgroup analysis of the GC patients also revealed that patients with 19q_Amp (HR = 8.93; 95% CI, 1.44‐55.30; *P* = .005; Figure 3C and Table 2) and 9p_Del had worse PFS (HR = 3.15 × 10^9^; 95% CI, 0‐Inf; *P* < .001; Figure 3D and Table 2), and lower ORRs than those without the respective alterations (19q_Amp, 0% vs 44.4%; 9p_Del, 0% vs 40.0%; Figure S1E,F).

### 3.7 TMB and CIS correlated with clinical benefit to immunotherapy

**FIGURE 4:**
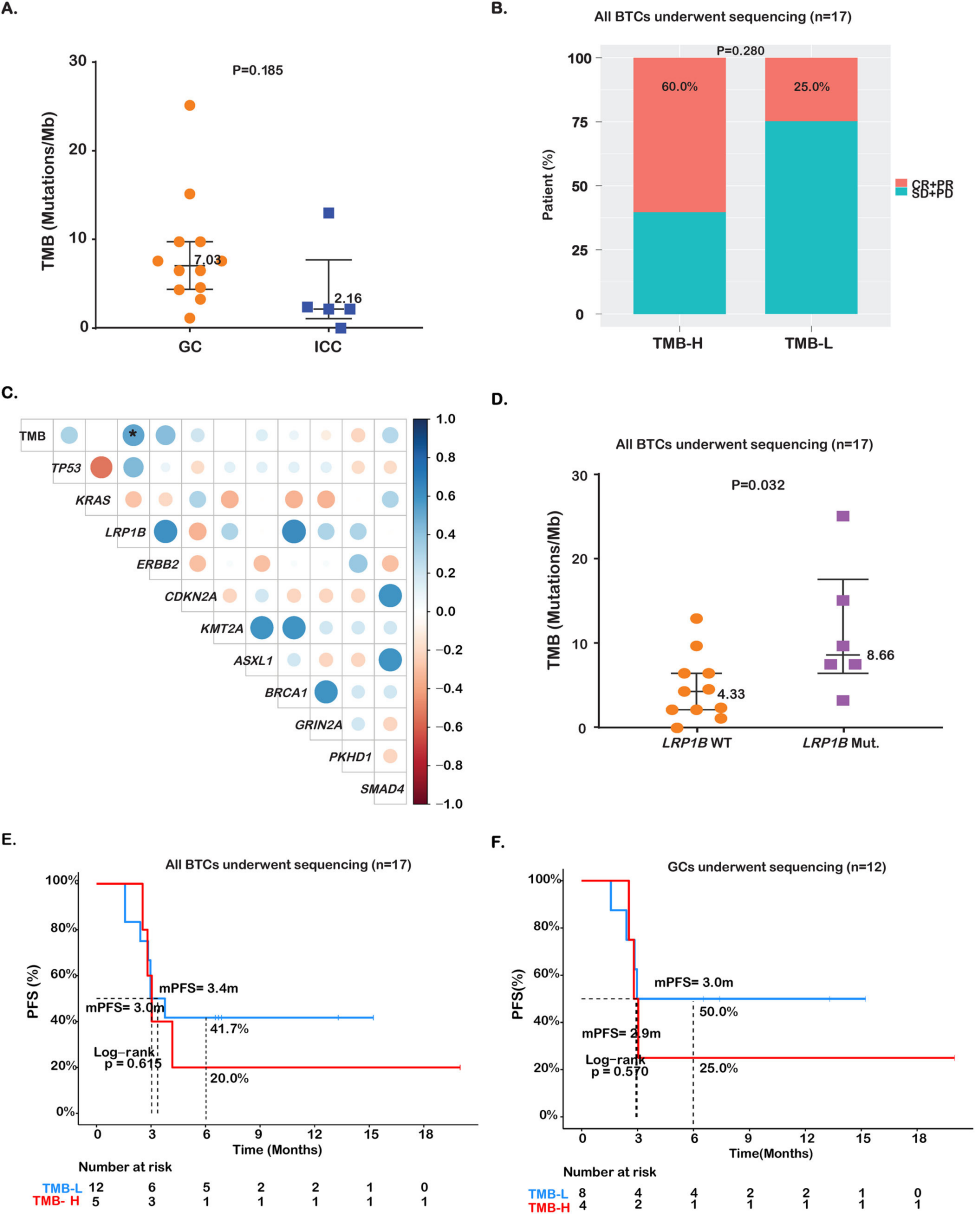
Associations of TMB with immunotherapy outcome. A, Comparison of TMB between samples with GCs and intrahepatic ICCs. **B,** Comparison of ORR in the TMB‐H and TMB‐L subgroups of all BTCs (n = 17). **C,** Correlation analysis among genes altered in BTCs and with TMB (n = 17). **D,** Comparison of TMB between *LRP1B* wildtype and mutant BTCs in BTCs (n = 17). **E,F,** PFS comparison between the patients with TMB‐H and TMB‐L in BTCs (n = 17) or GCs (n = 12), respectively

Finally, we evaluated several genome instability‐related features, including MSI, TMB, and chromosomal instability, which are often indicative of a high neo‐antigen burden and consequently response to immunotherapy. Consistent with the rare occurrence of MSI in BTCs, all 17 patients with evaluable NGS results were microsatellite stable. TMB was higher in GCs samples than those with ICCs (median TMB, 7.03 vs 2.16 mut/Mb; *P* = .19; Figure 4A). Although no survival advantage was observed in BTCs with TMB‐H than those with TMB‐L (Figure 4E), a high proportion of them experienced objective response (TMB‐H vs TMB‐L, 60.0% vs 25.0%; Figure 4B). In GCs, we also found no difference in PFS between patients with TMB‐H and TMB‐L (Figure 4F), but a higher ORR in TMB‐H patients (TMB‐H vs TMB‐L, 50.0% vs 25.0%; data not shown).

Furthermore, correlation analysis of TMB with genetic alterations in all BTCs revealed a strong correlation between *LRP1B* mutations and TMB (*P* = .02; Figure 4C). Indeed, TMB was significantly higher in patients with *LRP1B* mutations (median TMB, 8.66 vs 4.33 mut/Mb; *P* = .032, Student's *t*‐test; Figure 4D).

**FIGURE 5:**
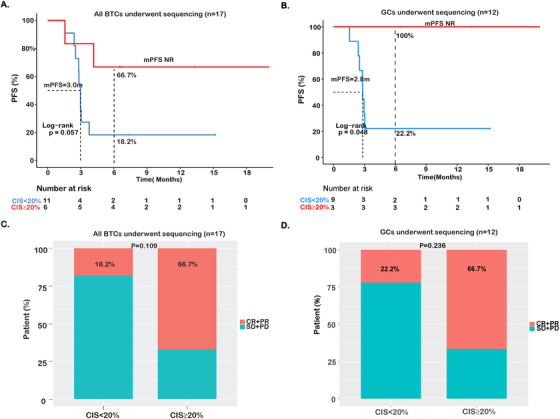
Associations of chromosomal instability with immunotherapy outcome. **A,B,** Kaplan‐Meier estimates of PFS comparing the subgroups with (chromosomal instability score, CIS > = 20%) and without (CIS < 20%) chromosomal instability in BTCs (n = 17) or GCs (n = 12), respectively. **B,** Comparison of ORR in the subsets with and without chromosomal instability in BTCs (n = 17) or GCs (n = 12), respectively

On the other hand, BTC patients with chromosome instability, as determined by the CIS of 0.2 or higher, showed improved PFS outcome (mPFS = NR vs 3.0 months; HR = 0.24; 95% CI, 0.05‐1.17; *P* = .058; Figure 5A), as well as increased ORR (66.7% vs 18.2%; Figure 5C). For GCs, patients with higher CIS displayed significantly better survival (mPFS = NR vs 2.8 months; HR = 9.4 × 10^10^; 95% CI, 0‐Inf; *P* = .048; Figure 5B), and also had a higher ORR (66.7% vs 22.2%; Figure 5D).

**FIGURE 6:**
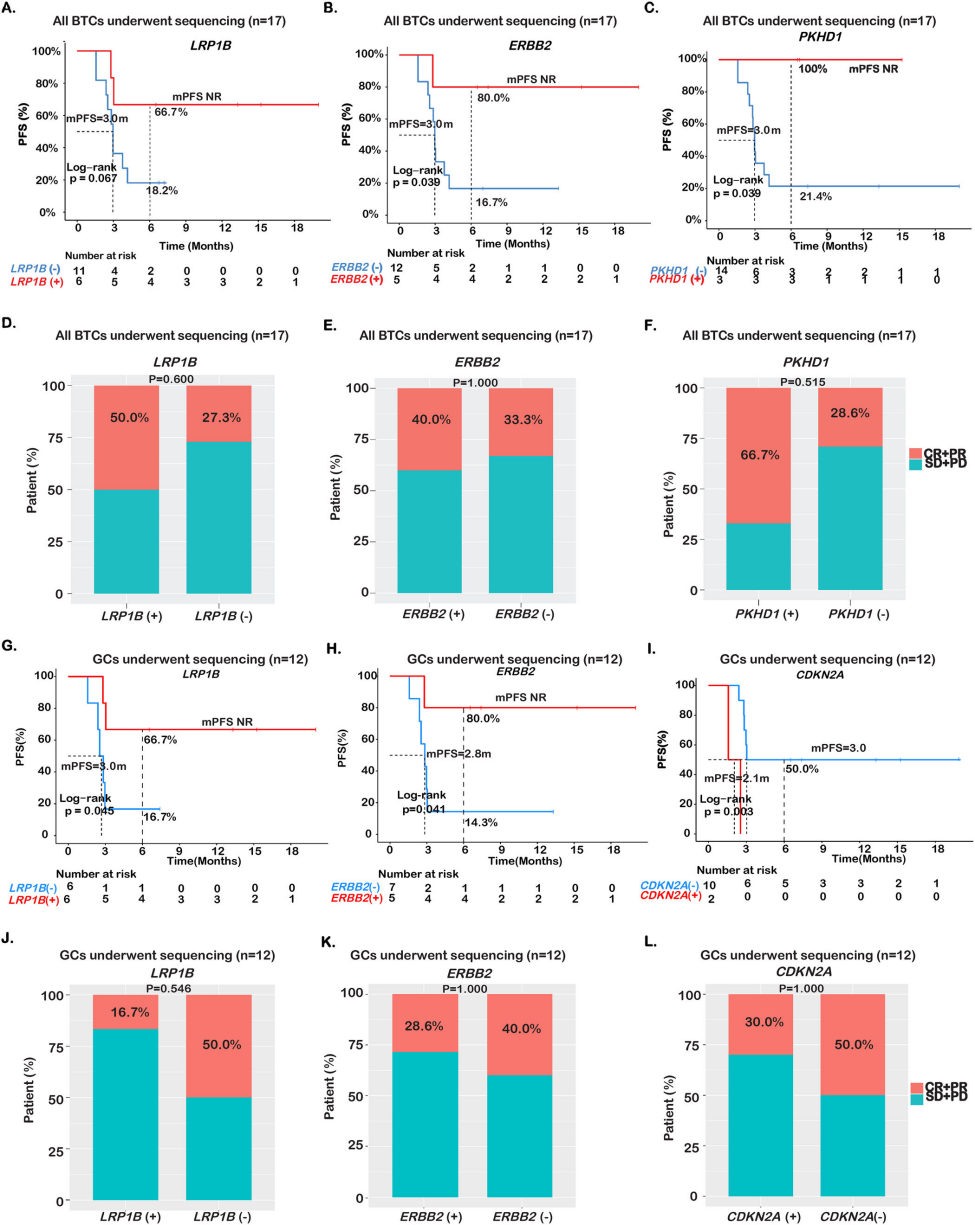
Effect of gene (**A, D**) *LRPB1*, (**B, E**) *ERBB2*, and (**C, F**) *PKHD1* mutations on patient PFS and ORR in BTCs (n = 17), and effect of gene (**G, J**) *LRPB1*, (**H, K**) *ERBB2*, and (**I, L**) *CDKN2A* mutations on patient PFS and ORR in GCs (n = 12)

## 4 DISCUSSION

While chemotherapy is considered as the standard option for advanced BTCs, the prognosis remains poor and no standard second‐line therapy has been established following disease progression. With limited treatment options and the aggressive disease course, immune checkpoint blockade represents a promising therapeutic choice for advanced BTCs patients. Herein, we evaluated the efficacy and safety of ICI treatment alone or in combination with other antitumor therapies, and provided clinical evidence for the first time for the predictive value of potential genomic correlates of immunotherapy response in patients with advanced disease.

KEYNOTE‐028 and KEYNOTE‐158 are two basket trials that examined the efficacy and safety of Keytruda in advanced BTCs patients who progressed on first‐line standard treatment regimens.^11^ In these two studies, pembrolizumab treatment is associated with durable clinical activity and manageable toxicity. In KEYNOTE‐028, the ORR was 13% with an mPFS of 1.8 months and an mOS of 6.2 months. In KEYNOTE‐158, the ORR was 5.8% with an mPFS of 2.0 months and an mOS of 7.4 months. Another phase I study also demonstrated durable clinical activity of nivolumab in advanced BTCs patients.^29^ In patients who were refractory to standard chemotherapy, the mPFS was 1.4 months, mOS was 5.2 months, and ORR was 3.3%. In those who received nivolumab and chemotherapy combination as first‐line treatment, the mPFS was 4.2 months, mOS was 15.4 months, and ORR was 36.7%. Other immune‐combination therapies have been evaluated in the clinic. One phase II study of an anti‐angiogenic drug, lenvatinib, combined with ICI in 14 ICCs who had received at least two prior anticancer therapies reported a 21.4% ORR and 5.9 months mPFS.^30^ On the other hand, a phase Ib study of pembrolizumab plus ramucirumab (another anti‐angiogenic drug) on patients who progressed first‐line therapies, reported a 4% ORR, 1.6‐month mPFS, and 6.4‐month mOS.^31^

Our study cohort composed of advanced BTC patients who received ICI treatment as first‐line or following progression after prior standard treatment regimens. Most of the patients enrolled in our study received ICI treatment combined with gemcitabine‐based chemotherapy, and the rest received ICI treatment either as monotherapy or in combination with anti‐VEGFR agents. The antitumor activity of ICI in our mixed cohort was in line with previous reports with an mPFS of 3.4 months, mOS of 8.0 months, and ORR of 26.9%, as well as a manageable toxicity profile.

The search for potential markers that enrich for responders to immunotherapy is one intense area of research. While patients with MSI tumors might benefit from immunotherapy, the proportion of MSI BTCs is very low as demonstrated by studies from our group and others.^32^ In the exploratory analysis of the KEYNOTE‐028 study, it was found that TMB and/or a T‐cell inflamed gene‐expression profile is predictive of immunotherapy response across 20 cancers.^10^ Systemic inflammation plays an important part in tumorigenesis and immune evasion. Indeed, several inflammatory markers have been described as prognostic factors in cancer patients.^33^ Herein, we also assessed a wide array of systemic inflammatory markers, including LDH, CRP, SII, GPS, PI, PNI, as well as several tumor markers, including CEA, CA125, and CA199. However, none of them showed a clear association with immunotherapy outcome. As BTCs are associated with chronic inflammation, it is possible that there might be adaptive mechanisms in which patient outcome is not highly dependent on the inflammatory microenvironment. Our study also assessed the association between TMB and clinical outcome. One previous study showed that the median number of nonsilent somatic mutations were higher in GCs compared with ICCs or extrahepatic cholangiocarcinoma^34^ similar to what we demonstrated in this study. While TMB was not statistically predictive for PFS outcome, we did notice a higher response rate to immunotherapy of BTC patients with high TMB.

To the best of knowledge, no extensive analyses that correlate genomic characteristics with immunotherapy response have been carried out in BTCs. In our cohort, we looked at genomic alterations in ICI‐treated BTC patients at the levels of single nucleotide variations, CNVs, signaling pathways, and chromosomal aberrations. Similar to TMB, chromosomal instability might also reflect the neoantigen load in the tumor. As expected, we found that patients with high CIS were associated with improved PFS and ORR. At the single‐gene level, our results showed better PFS in patients with *ERBB2*, *LRP1B*, and *PKHD1* alterations. Consistent with previous reports,^34^, ^35^ we also found higher prevalence of *ERBB2* alterations in patients with GCs than in ICCs. Yet the functional significance of *ERBB2* missense mutations identified in our study cohort remained unclear. In addition, the association between *ERBB2* and improved immunotherapy outcome might be confounded the presence of additional alterations in genes including *PKHD1*. Thus, the role of *ERBB2* in promoting the antitumor effect of ICIs in BTCs is debatable. Mutations in *LRP1B*, the low‐density lipoprotein (LDL) receptor family gene, have been associated with high TMB and improved immunotherapy outcome in melanoma and NSCLC cancer.^36^ Cases with *LRP1B* mutations were characterized by an enrichment of genes involved in cell cycle checkpoints and antigen processing and presentation.^36^ In 2020 ASCO, one multicenter study shown that independent of TMB/MSI status, patients with pathogenic *LRP1B* alterations could achieve impressive and durable objective response rates to ICI.^37^ In our study, the result also suggested that genetic mutations of *LRP1B* showing higher TMB and predicted for favorable immunotherapy response in BTCs. In addition, our study suggests *PKHD1* as a potential biomarker for better immunotherapy response. The association between *PKHD1* mutations and immunotherapy outcome might be due to its effect on the immune system. Studies have shown that *PKHD1* mutations are responsible for congenital hepatic fibrosis and autoimmune cholangitis.^38^, ^39^ PKHD1 mediates secretion of chemokines in cholangiocytes, and consequently the recruitment of macrophages or immune cell infiltration.^39^ However, given the low prevalence of *PKHD1* mutations in our cohort, future evidence is required to support its association with immunotherapy.

The effect of arm‐level SCNAs on immunotherapy response may be attributed to the changes in the dosage of genes with immune‐modulating effects. Our study suggests 19q amplification and 9p deletion as potential markers of poor immunotherapy outcome. Chromosome 19q contains *TGFB1*, which plays a role in immune cell recruitment and the pathogenesis of chronic inflammatory conditions.^27^, ^40^ Chromosome 9p contains CD274 (encoding PD‐L1) and multiple interferon genes, including *IFNA1*, *IFNA2*, that are involved in macrophage polarity and immune checkpoint regulation.^27^

In summary, results from this study and others support the use of immunotherapy alone or in combination with other anticancer drugs in advanced BTCs patients. We also provided preliminary clinical data for the first time showing the predictive potential of genomic correlates of immunotherapy response in advanced BTCs. However, due to the small number of samples and the clinical heterogeneity of the study cohort, the prognostic value of these genetic biomarkers needs to be interpreted carefully. Further larger‐cohort studies are necessary to confirm the efficacy of immunotherapy in advanced BTCs and the predictive value of relevant biomarkers.

## CONFLICT OF INTEREST

Xiaoying Wu, Mengmeng Wu, Fufeng Wang, Junrong Yan, Jiachen He and Yang W. Shao are the employees of Nanjing Geneseeq Technology Inc. The other authors report no conflict of interest.

## AUTHOR CONTRIBUTIONS

Study concept and design: Jieer Ying and Wei Wu. Analysis and interpretation of data: Jingjing Li, Xiaoying Wu, Mengmeng Wu, Fufeng Wang, Junrong Yan, Jiachen He and Yang W. Shao. Clinical information colllection: Qing Wei, Jun Sima, Haibo Mou, Hanguang Hu, Jianguo Zhao, Da Li, Jinlin Hu, Lingnan Zhang, Lei Chen and Cong Luo. Pathological evaluation: Xiu Zhu. The dataset used during the study are available from the authors of the study on a reasonable request. All authors were involved in the drafting, review, and approval of the report and the decision to submit for publication.

5

## Supporting information

SUPPORTING INFORMATION

SUPPORTING INFORMATION

## Data Availability

The dataset used during the study are available from the authors of the study on a reasonable request.
