# Supplementary figures and images for: Integrative clinical and molecular analysis of advanced biliary tract cancers on immune checkpoint blockade reveals potential markers of response
Source: Clin Transl Med. 2020 Aug 12;10(4):e118. doi: 10.1002/ctm2.118 (PMC7423188; doi:10.1002/ctm2.118)

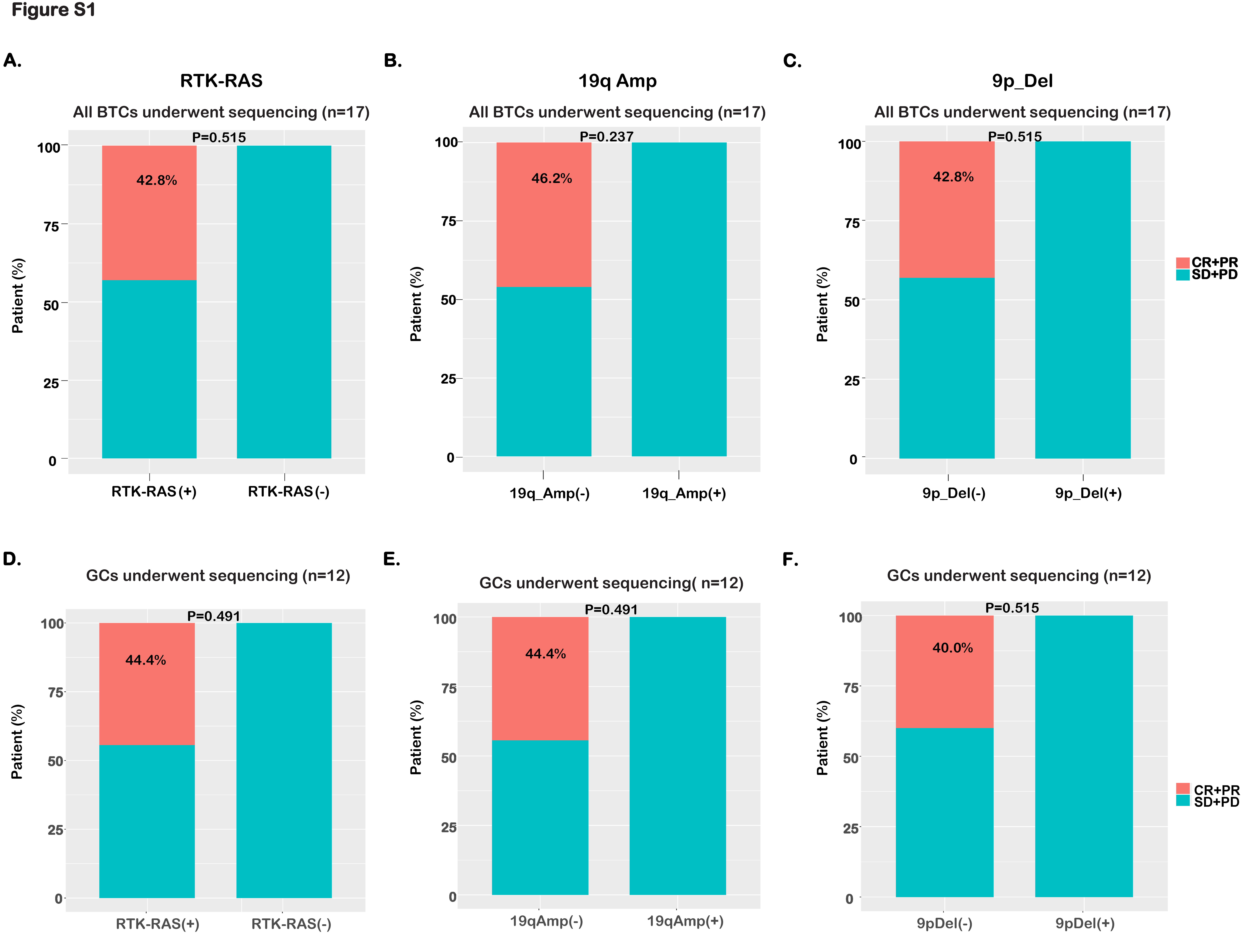

Supplement: Supplementary file 1 — SUPPORTING INFORMATION [file CTM2-10-e118-s002.tiff]
